# Genomic screen for loci associated with tobacco usage in Mission Indians

**DOI:** 10.1186/1471-2350-7-9

**Published:** 2006-02-10

**Authors:** Cindy L Ehlers, Kirk C Wilhelmsen

**Affiliations:** 1 Departments of Molecular and Experimental Medicine, and Neuropharmacology, The Scripps Research Institute, La Jolla California, USA; 2 Departments of Genetics and Neurology, The Carolina Center for Genome Sciences and the Bowles Center for Alcohol Studies, University of North Carolina, North Carolina, USA

## Abstract

**Background:**

The prevalence of tobacco usage in Native American adults and adolescents is higher than any other racial or ethnic group, yet biological risk and protective factors underlying tobacco use in this ethnic group remain unknown. A genome scan for loci associated with tobacco use phenotypes was performed with data collected from a community sample of Mission Indians residing in Southwest California.

**Methods:**

A structured diagnostic interview was used to define two tobacco use phenotypes: 1) any regular tobacco usage (smoked daily for one month or more) and 2) persistent tobacco usage (smoked at least 10 cigarettes a day for more than one year). Heritability was determined and a linkage analysis was performed, using genotypes for a panel 791 microsatellite polymorphisms, for the two phenotypes using variance component methods implemented in SOLAR.

**Results:**

Analyses of multipoint variance component LOD scores for the two tobacco use phenotypes revealed two scores that exceeded 2.0 for the regular use phenotype: one on chromosomes 6 and one on 8. Four other loci on chromosomes 1,7,13, and 22 were found with LOD scores between 1.0 and 1.5. Two loci of interest were found on chromosomes 1 and 4 for the persistent use phenotype with LOD scores between 1.3–1.5. Bivariate linkage analysis was conducted at the site on chromosome 4 for persistent tobacco use and an alcohol drinking severity phenotype previously identified at this site. The maximum LOD score for the bivariate analysis for the region was 3.4, however, there was insufficient power to exclude coincident linkage.

**Conclusion:**

While not providing evidence for linkage to specific chromosomal regions these results identify regions of interest in the genome in this Mission Indian population, for tobacco usage, some of which were identified in previous genome scans of non-native populations. Additionally, these data lend support for the hypothesis that cigarette smoking, alcohol dependence and other consumptive behaviors may share some common risk and/or protective factors in this Mission Indian population.

## Background

Tobacco use by North American Indians was documented by the first European explorers to the continent who described it as being consumed by smoking, chewing or in salves [1,2]. Early Indian tobacco usage appeared to be primarily for ceremonial purposes, and it has been suggested that older forms of tobacco were more potent and may have contained other psychoactive substances [3]. Currently, smoking rates among North American Indians are higher than any other racial or ethnic group, despite significant variation across tribes and regions of Canada and the US [4-8].

Smoking has been described as particularly prevalent in Native American youth. No differences have been found between rural and urban Native American adolescents in one study of smoking prevalence, and in that study Native American youth reported more exposure to peers who smoked and greater access to cigarettes than other racial and ethnic groups [9]. American Indian middle school students report initiating smoking with family, friends and/or peers and also report no links between the initiation of recreational smoking and the use of tobacco in traditional ceremonies [10].

Psychosocial variables such as death, loss, and other stressful life events have been demonstrated to be risk factors for American Indian adolescents initiating smoking [11], however, such variables do not account for all of the variance associated with increased risk in this ethnic group. Studies of smoking behavior in non-native populations have revealed that smoking-related traits are complex and that genetic variables significantly influence smoking behaviors [12-14]. There also appears to be some shared use liability between tobacco and others drugs [15-18]. Several studies have identified regions in the genome that are associated with tobacco related phenotypes [19-26]. Most of the studies have published on one of four population cohorts: the Collaborative Study on the Genetics of Alcoholism, the Christchurch New Zealand sample, a family study of panic disorder, and the Framingham Heart Study. The phenotypes in these studies have varied and the linkage analyses have not appreciably converged on any single genetic loci. No linkage analyses have as yet been published in a North American Indian population. American Indians are a unique population to study tobacco use phenotypes because of the presumption of relative genetic homogeneity and possibly more uniform exposure to environmental factors that could improve power in linkage analyses.

The present report is part of a larger study exploring risk factors for substance dependence among Native American Mission Indians [27-34]. The lifetime prevalence of alcohol and illicit drug dependence in this Indian population is high, and evidence for heritability of substance dependence has been demonstrated [31,35]. The purpose of the present set of analyses was to determine the heritability of tobacco use phenotypes in Mission Indian families and to identify genetic loci associated with those phenotypes. These data are also discussed in the context of previously published genome scans for tobacco use phenotypes in non-Indian populations as well as data demonstrating linkage to alcohol use disorder phenotypes in this population of Mission Indians [31,36].

## Methods

Participants, known collectively as Mission Indians, were recruited using a combination of a venue-based method [37,38] and a respondent-driven procedure [39] from eight geographically contiguous reservations in Southwest California as described previously [30,31]. Approval for the study was obtained from both The Scripps Research Institute Internal Review Board, and The Indian Health Council Board. Potential participants first met individually with research staff to have the study explained, give written informed consent, and respond to a screening questionnaire that was used to gather information on demographics, personal medical history, ethnicity and detailed measures of substance abuse history [40]. Each participant also completed an interview with the Semi-Structured Assessment for the Genetics of Alcoholism (SSAGA) [41] which was used to make drug dependence diagnoses [42]. The SSAGA is a fully structured, poly-diagnostic psychiatric interview that has undergone both reliability and validity testing [41,43]. It has been successfully used in Native American populations previously [44,45]. The interview retrospectively asks about the initial and regular use and problems associated with the use of drugs. The SSAGA I interview also allows for the diagnoses of drug abuse and dependence, but not for tobacco. Therefore, two tobacco use phenotypes were constructed based on individual SSAGA items. The first was regular tobacco use defined as: smoking daily for a month or more; and the second was persistent smoking defined as: smoking 10 or more cigarettes a day for more than a year.

One hundred pedigrees containing 885 individuals were used in the analyses. Of these, 244 individuals have both genotype and phenotype data and 222 additional individuals have only phenotypic data (total of 466). Fifty-nine families have only a single individual with phenotype data. These individuals were included within some analyses to the extent that they contribute information about trait means and variance and the impact of co-variants. The family sizes for the remaining families ranged between 4 and 38 subjects (average 13.5 ± 10) with between 2 and 15 individuals having both genotype and phenotype data (average 5.4 ± 4.2). Forty-one families were genetically informative. The data includes: 77 parent-child, 212 sibling, 26 half sibling, 8 grandparent-grandchild, 151 avuncular, and 245 cousin relative pairs where both genotype and phenotype data were available for both pair members. These data analyses have been described previously [31,36].

DNA was isolated from whole blood using an automated DNA extraction procedure, genotyping was done as previously described [46]. Genotypes were determined for a panel 791 autosomal microsatellite polymorphisms [47] using fluorescently labeled PCR primers under conditions recommended by the manufacturer (HD5 version 2.0; Applied Biosystems). The HD5 panel set has an average marker-to-marker distance of 4.6 cM, and an average heterozygosity of greater than 77% in a Caucasian population. Allele frequencies were estimated from genotype data for the entire population.

Genotypes were ultimately determined for 243 subjects. The total additive genetic heritability (H^2^) and its standard error were estimated for the regular and persistent tobacco use phenotypes using SOLAR. Variance component estimate methods were used to calculate LOD scores using SOLAR v2.0.4 [48,49] Genhunter 2 [50] and Merlin [51] with similar results. Simulation analysis was used to estimate empirical LOD scores and make appropriate genome wide adjustments for non-normality when the trait was modeled as normally distributed trait [52]. Gender was not a significant covariate for either phenotype. Age accounted for 1% of the phenotypic variance for persistent tobacco usage. Linkage analysis results were not significantly affected by exclusion of age as a covariate.

Two variance component analytic approaches were used for the traits. In the first approach the trait was modeled as a latent normally distributed variable with a threshold above which an individual is considered "affected". Using a second approach, the trait was modeled as a normally distributed variable with a correction for non-normality based on simulation. For these traits the LOD score was higher throughout the genome for the latent threshold model. The figures show the slightly lower LOD analysis treating the traits as continuous variable with simulations to correct for normality. These methods can occasionally give very dissimilar results presumably because of factors related to convergence. In this analysis it was required that both methodologies provide support for linkage. All results presented were derived from the use of SOLAR.

Bivariate analyses were conducted with data on chromosome 4 for persistent tobacco use where a loci was identified in a location at @102 cM where we had previously identified a linkage signal for alcohol drinking severity [31]. Drinking severity was defined using items in the SSAGA. Briefly, the SSAGA groups individual interview response items into nine categories that correspond to the nine DSMIII-R criteria used for making an alcohol dependence diagnosis. These items include: alcohol use severity items, legal, family, work and medical problems, tolerance, wanting/unable to quit and withdrawal. Of these potential phenotypes, alcohol use severity and alcohol withdrawal were found to be highly heritable. The alcohol severity phenotype grouped responses on four alcohol use severity items: 1) drank more than intended/more days in a row or when promised self wouldn't for three or more times, 2) drunk when didn't want to three or more times, 3) during drinking or recovering from the effects of drinking had little time for anything else, and 4) given up or greatly reduced important activities to drink. Each subject was scored as having 0–4 alcohol severity symptoms. The bivariate analysis using the persistent smoking phenotype and the alcohol use severity phenotype was performed using SOLAR (2.1.4)[49]. To test for pleiotropy, by excluding coincident linkage, the method proposed by Almasy [53] and as elaborated by North [54], was used. Briefly, the likelihood for the linkage model in which *p*_*q *_was estimated was compared to the likelihood for the linkage model in which *p*_*q *_was constrained to 1 (or -1 complete pleiotropy) or 0 (complete coincident linkage).

## Results

The demographic characteristics of the sample are virtually equivalent to the U.S. census data for the tribe, and have been presented previously [31]. Participants in the study were between 18 and 60 yrs with a mean age of 30 yrs (0.54), 58% were female, they had a mean education level of 11 (0.12) yrs and their income was $27,000 (0.11). The number of individuals with a Native American heritage of greater than 50% was 53% in the larger sample and 55% in the linkage sample. Indian heritage was based on their federal Indian blood quantum. The self-reported racial/ethnic admixture was primarily: Hispanic, Spanish, and other European. The linkage sample did not differ from the larger sample on any demographic or phenotypic variables.

Two hundred seventy-one out of a larger sample of 466 participants (58%) met the criteria for regular tobacco usage and (30%) met criteria for persistent usage. Men were not significantly more likely to be regular users or persistent users. Nor were those participants having a native heritage of 50% or greater likely to be regular or persistent users compared to those with lesser degrees of Native heritage. The mean age of onset of regular use was 17 yrs and the mean quantity and frequency of usage was 12 cigarettes a day for 9 years. Persistent and regular smokers did not differ from each other or non-smokers on age, education or income. The prevalence of alcohol dependence in the larger sample was 60% and in the linkage sample it was 61%. Alcohol dependence was co-morbid with both the persistent (Chi square = 12.67; df = 1; p < 0.0001) and regular (Chi square = 22.60; df = 1; p < 0.00001) smoking phenotypes.

The first aim of the study was to determine the heritability of the two tobacco phenotypes. The estimated heritability (h^2^) for the regular tobacco usage, treating the phenotype as a continuous variable, was 0.37 ± 0.11 and persistent tobacco usage, 0.34 ± 0.12. Estimated heritability nominally increased to 0.53 ± 0.17 and 0.46 ± 0.18 when a latent threshold model was used.

**Figure 1:**
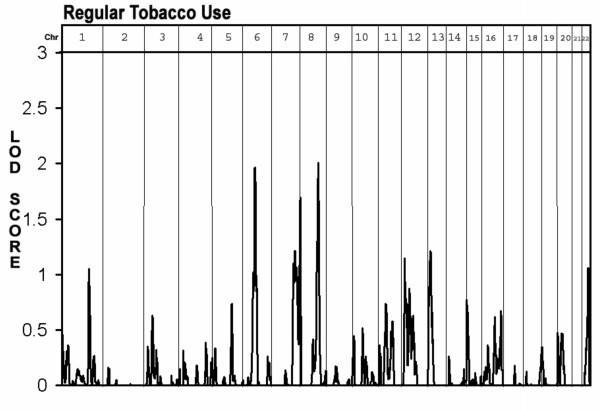
Multipoint Linkage Analysis for the "regular tobacco use" phenotype for the entire genome. Results for each chromosome are aligned end to end with the p terminus on the left. Vertical lines indicate the boundaries between the chromosomes. The numbers above on the X-axis indicate the chromosome number.

**Figure 2:**
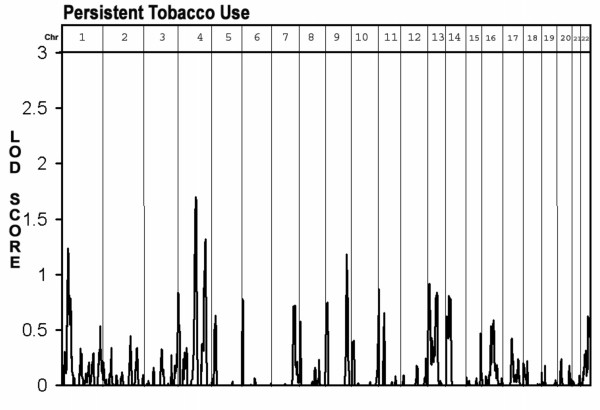
Multipoint Linkage Analysis for the "persistent tobacco use" phenotype for the entire genome. Results for each chromosome are aligned end to end with the p terminus on the left. Vertical lines indicate the boundaries between the chromosomes. The numbers above on the X-axis indicate the chromosome number.

The second aim of the study was to identify loci linked with the two tobacco use phenotypes. As seen in Figure 1 and table 1, loci associated with regular tobacco usage were found on chromosome 6 at 75 cM (between D6S257 and D6S460, LOD = 2.0) and on chromosome 8 at 115 cM (at D8S1784, LOD = 2.0). Additional "loci of interest" were found on chromosomes 1 at 175 cM (at D1S2878, LOD = 1.2) chromosome 7 at 181 cM (at D7S2465, LOD = 1.7) chromosome 13 at 15 cM (D13S217 LOD = 1.3) and chromosome 22 at 45 cM (between D22S423 and D22S274, LOD = 1.3). As seen in figure 2 and table 1, only two "loci of interest" were found for the persistent tobacco use phenotype, one on chromosome 1 at 40 cm (near D1S199, LOD = 1.3) and one on chromosome 4 at 102 cM (near D4S414, LOD = 1.5). No overlaps were found between loci identified for the regular use phenotype and those found for the persistent use phenotype.

**Table 1:** Chromosome Locations

CHR	Trait	LOC (cM)	LOD	Nearest Marker	Supporting References (phenotype)
1	Persistent Tob Use	75	1.3	D1S2652	
1	Regular Tob Use	175	1.2	D1S196	Bierut et al. 2004 (Alc dep & smoking)
4	Persistent Tob Use	100	1.5	D4S2460	Straub et al. 1999, Duggirala et al. 1999 (Tob), Long et al. 1998 (Alc dep), Reich et al. 1998 (Alc dep), Saccone et al. 2003 (Max drinks), Ehlers et al. 2004b(Alc drink severity)
6	Regular Tob Use	50–75	2.0	D6S1575	Ehlers et al. 2004b (Alc withdrawal), Bergen et al. 1999 (Smoking)
7	Regular Tob Use	140	1.5	D7S640	Gelernter et al. 2004
8	Regular Tob Use	110	2.0	D8S1762	
13	Regular Tob Use	20	1.3	D13S289	Saccone et al. (2003)
22	Regular Tob Use	40–50	1.3	D22S274	Saccone et al. (2003)

Abbreviations: CHR (chromosome number), LOC (location), Tob Use (tobacco usage), Alc dep (Alcohol dependence), Tob (Tobacco use phenotypes), Max drinks (maximum number of drinks consumed in a 24 hr period), Alc withdrawal (alcohol withdrawal severity phenotype) Alc drink severity (severity of drinking symptoms from DSM-III-R alcohol dependence).

**Figure 3:**
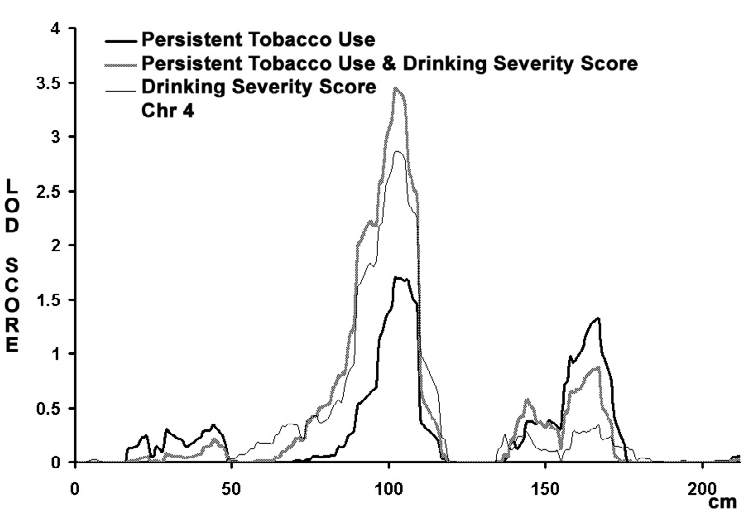
Multipoint, univariate and bivariate analysis for chromosome 4. Univariate analysis of the "persistent tobacco use" phenotype is shown in the heavy dark line and univariate analysis for the "drinking severity" phenotype in the thin line. The bivariate analysis of the "persistent tobacco usage and drinking severity" is shown in the dark line. The analysis assumes a latent normally distributed variable with a threshold above which an individual is affected. The maximum LOD score for the bivariate analysis for the region is 3.4 compared to a maximum of 2.8 for drinking severity score and 1.7 for persistent tobacco use. Although there was insufficient power to exclude coincident linkage (p = 0.26), complete pleiotropy could be excluded (p = 0.02).

The peak LOD score observed for persistent tobacco use at 102 cM on chromosome 4 coincides with LOD score peak previously reported in this Mission Indian sample for a drinking severity phenotype [31] (LOD score 2.9). The region near 100 cM where the peak LOD score was observed is notable because it contains a cluster of genes that code for alcohol dehydrogenases (ADH) and has been implicated in several studies of alcohol and tobacco use. Table 1 lists the loci identified in the current study and also provides reference to findings from previously published studies in this Mission Indian population and other population samples. In order to test whether the current findings for drinking severity and persistent smoking are coincident on chromosome 4, bivariate linkage analysis was conducted (see Figure 3). The maximum LOD score for the bivariate analysis for the region is 3.4 compared to a maximum of 2.8 for drinking severity score and 1.7 for persistent tobacco use. Although there was insufficient power to exclude coincident linkage (p = 0.26), complete pleiotropy could be excluded (p = 0.02).

## Discussion

A variety of genetically influenced characteristics most likely contribute to the increased risk for tobacco usage seen in Mission Indians. A number of psychosocial variables have been associated with tobacco usage in Native Americans teens including life events and availability [9-11]. However, this is the first study to determine heritability of tobacco usage and to conduct a linkage analysis to tobacco related phenotypes in Native Americans. Regular and persistent tobacco use were both found to be moderately heritable as measured by h^2^. Additionally, several chromosomal loci were identified as regions of interest that may harbor tobacco usage genes.

There were two loci identified on chromosome 1, one for persistent tobacco usage at 75 cM, and one for regular tobacco usage at 175 cM. Both of these loci were identified in a genome scan conducted using the Collaborative Study of the Genetics of Alcoholism (COGA) dataset for habitual smoking and alcohol dependence [19]. Loci on chromosome 1 have been identified previously for alcohol dependence phenotypes in a number of studies [55-60]. However, using the COGA dataset, Bierut [23] demonstrated the combined phenotype of alcohol dependence and habitual smoking had allele sharing among sibling pairs that was similar to results for alcohol dependence alone. Additionally, no evidence for linkage to alcohol dependence has been found at that location on chromosome 1 in this Mission Indian population [31]. Taken together these results suggest that there may be specific genetic factors impacting tobacco usage that do not impact alcohol dependence within this support interval on chromosome 1.

One of the most consistent findings between several genetic linkage studies conducted in Native American and other mixed heritage populations is evidence for a protective association for alcohol dependence and related behaviors in a region on chromosome 4 near the Alcohol dehydrogenase gene cluster. In a genetic linkage study evaluating large families who were members of a Southwest Indian tribe, three loci in this region on chromosome 4q showed evidence for linkage [61]. Evidence for linkage at this site, in Mission Indians, was also identified in a genome scan for a severity of alcohol drinking phenotype [31]. A protective association has also been found between polymorphisms in ADH1B3, alcohol dependence, and tobacco usage in this population [34]. Additionally both the "unaffected by alcoholism" [55] and "maximum drinks ever consumed in a 24 hour period" [62], phenotypes, were found to give evidence for linkage on chromosome 4 in the region of the ADH gene cluster in the COGA study. Evidence for linkage to tobacco dependence at this same general region of the genome on chromosome 4 has also been demonstrated in both the Richmond and Christchurch samples described in the publications by Straub [22], and by Duggirala [63]. The linkage analysis for persistent tobacco use in Mission Indians also supports these observations. The detection of a linkage signal for drinking severity using the same cohort in this same region suggests that there may be a sequence variant that affects substance abuse in general or that there are at least two tightly linked variants that affect different aspects of substance abuse behavior. The bivariate analysis done in these studies is not sufficiently powered to distinguish between these possibilities.

There is one additional locus of interest in this Mission Indian study that was also identified in previous linkage analyses for alcohol-related phenotypes. Evidence for linkage was found on chromosome 6 for the regular tobacco use phenotype in a support interval previously identified for an alcohol withdrawal severity phenotype in this Mission Indian population [31]. Additionally, evidence for an epidemiological measure of smoking was also found at this site using the COGA dataset [64].

Additional loci of interest were found on chromosome 7, 13 and 22 for regular smoking that have been identified in other studies [19,25]. Only a few findings appear to be replicated between studies. This is most likely related to a number of factors. Some of the studies utilized participants recruited from clinical populations enriched for psychiatric disorders whereas some were population based samples using mixed ethnicities. Also, differing definitions of smoking and tobacco dependence were used. More replications and additional studies in different ethnic groups are needed to further evaluate the linkage results [24].

The results of this study should be interpreted in the context of several limitations. First, the findings may not generalize to other Native Americans or represent all Mission Indians. Second, comparisons of linkage findings to non-Indian populations of drug abusers may be limited by differences in a host of potential genetic and environmental variables. Third, the underlying assumption that these phenotypes are normally distributed, an assumption of variance component analyses, may not be warranted. Finally, because this population has significant admixture estimates of allele frequencies may produce biased LOD scores. The linkage analyses do not give us any information on whether the genes within the loci identified are coding for protective or risk factors. Despite these limitations, this report represents an important first step in an ongoing investigation to understand the genetic determinants associated with the development of substance use disorders in this high risk and understudied ethnic group.

## Conclusion

These data represent the first family-based genome-wide chromosome segregation analyses for tobacco use phenotypes in Native Americans. The results corroborated the possible importance of several chromosomal regions highlighted in prior linkage studies for substance abuse phenotypes and identify new regions of the genome for this ethnic group and/or Mission Indians. A replicate study is underway to test the reliability of the findings. Additionally, these data lend support for the hypothesis that cigarette smoking, alcohol dependence and other consumptive behaviors may share some common risk and/or protective factors in this Mission Indian population.

## List of abbreviations

SSAGA, Semi-Structured Assessment for the Genetics of Alcoholism; H^2 ^total additive genetic heritability; LOD, log of the odds; COGA, collaborative study genetics of alcoholism; ADH, Alcohol Dehydrogenase.

## Competing interests

The author(s) declare that they have no competing interests.

## Authors' contributions

Cindy L Ehlers contributed to the recruitment, collection and analysis of the clinical and genetic data on the subjects. Kirk C. Wilhelmsen contributed to the analyses of family structures, genome scan, linkage and heritability analyses.

## Pre-publication history

The pre-publication history for this paper can be accessed here:


